# Data-mining analysis of media frame effects on social perception of schizophrenia renaming in Korea

**DOI:** 10.1186/s12888-023-05386-4

**Published:** 2023-11-27

**Authors:** Il Bin Kim, Joonho Choi, Seon-Cheol Park, Shinsuke Koike, Jun Soo Kwon, Eunkyung Kim, Hyo Sun Choi, Ju Yeon Lee, Yu Sang Lee

**Affiliations:** 1 Department of Psychiatry, CHA Gangnam Medical Center, CHA University School of Medicine, Gangnam, Republic of Korea; 2 Department of Psychiatry, Hanyang University Guri Hospital, Guri, Republic of Korea; 3 University of Tokyo Institute for Diversity and Adaptation of Human Mind (UTIDAHM), University of Tokyo, Tokyo, Japan; 4 Department of Psychiatry, Seoul National University College of Medicine, Seoul, Republic of Korea; 5 Medicine Center for Mental health research, Hanyang University College, Seoul, Republic of Korea; 6 Department of Psychiatry, Yong-In Mental Hospital, 940 Jungbu-daero, Giheung-gu, Yongin, Republic of Korea

**Keywords:** Schizophrenia, Johyeonbyung, Jungshinbunyeolbyung, Data-mining analysis, Social perception, Media frame

## Abstract

**Background:**

In 2011, Korean Neuropsychiatric Association renamed schizophrenia from ‘mind split disorder’ (‘Jungshinbunyeolbyung’ in Korean) to ‘attunement disorder’ (‘Johyeonbyung’ in Korean), in a strategic way to reduce social stigma toward people with schizophrenia. However, there remains an elusive consensus that how the renaming effort has contributed to changes in the social perception of schizophrenia in Korea.

**Methods:**

With this regard, we explored whether media frames alter the social perception, in ways of respecting or disrespecting schizophrenia patients before and after the renaming. This study extensively investigated media keywords related to schizophrenia across the time by applying both language and epidemiologic analyses.

**Results:**

In results, the media keywords have been negatively described for schizophrenia patients both before and after the renaming. Further, from an analysis using the regression model, a significant correlation was observed between the frequency of negative keywords and the hospitalization frequency of schizophrenia patients.

**Conclusions:**

These findings suggest that the social perception of schizophrenia has been scarcely changed, but rather remained negatively biased against schizophrenia patients, in spite of the renaming effort. Notably, the biased media frames have been demonstrated to negatively impact on the social perception, and even on the medical use patterns of general schizophrenia patients. In conclusion, we suggest that the unbiased media frames along with the renaming effort may collectively help reduce the negative social perception of schizophrenia.

**Trial registration:**

This study was approved from the Institute of Review Board (IRB) of the Yoing-In Mental Hospital (IRB No. YIMH-IRB-2019-02).

**Supplementary Information:**

The online version contains supplementary material available at 10.1186/s12888-023-05386-4.

## Introduction

 One of the main goals in the psychiatry field has been to reduce a negative consequence such as discrimination from the stigmatization against patients affected by serious mental disorders, especially schizophrenia [1–3]. The stigmatization is widely recognized to illicit an adverse impact on the patients with schizophrenia, such in part as a delay or non-engagement in diagnosis and treatment for the disease. In order to reduce social stigma toward people with schizophrenia, Korean Neuropsychiatric Association renamed schizophrenia from ‘Jungshinbunyeolbyung’ to ‘Johyeonbyung’ in Korean in 2011. Previous studies that investigated the renaming effect on the social stigma of schizophrenia have yielded inconsistent results across countries [4, 5]. Still, there remains elusive consensus that how the renaming effort has contributed to improvement in the social perception of schizophrenia in Korea.

Not only naming of schizophrenia, media frame also effects on social perception of schizophrenia. In particular, negative images related to the perilous and violent trait of schizophrenia patients appear to be most prevalent [6–8]. Amounting literatures indicate the media frames perpetuate the negative stereotypes of the schizophrenia patients [9–13]. Indeed, media reports on patients with schizophrenia seem to deal frequently with acts of violent crimes, creating negative social perception of individuals with schizophrenia [14–16]. The media frame has been accordingly recognized to hold a considerable effect on the social stigma of schizophrenia patients. Thus, the social stigmatization against schizophrenia patients requires to be evaluated with regard to social media frames that putatively influence the public perception of schizophrenia.

This study targeted news articles related to schizophrenia (i.e. mind split disorder as ‘Jungshinbunyeolbyung’ in Korean before the renaming and attunement disorder as ‘Johyeonbyung’ in Korean after the renaming) to identify how the perception of public, which may produce a social pressure that hinders early diagnosis and continuous treatment of schizophrenia patients, has changed from the revision of the disease name. The research questions for this purpose are as follows: <A > How has the social perception of schizophrenia has changed before and after the disease renaming event in 2011? <B > What is the difference between the social perceptions of mind split disorder as ‘Jungshinbunyeolbyung’ (before the renaming of schizophrenia) and that of attunement disorder as ‘Johyeonbyung’ (after the renaming of schizophrenia)? <C > How is the association between the hospital admission patterns of schizophrenia patients and frequencies of media-reported negative keywords deciphering schizophrenia patients? Comprehensively, this study would help establish a desirable direction for media reporting stance and also contribute to creating a fair therapeutic environment for patients with schizophrenia.

## Methods

### Data

#### Online news articles

Online news articles were in advance collected to explore media reports and frames that reflect the social perception of schizophrenia patients. We specifically targeted the words of the news articles reported by about 800 media companies, which were comprehensively provided by the Korea’s gigantic online searching engine ‘Naver.com’, during a study period ranging from January 1, 2005 to December 31, 2018. Naver.com is the Korean media brand with the highest domestic utilization rate of above 65%, and the average political inclination of users encompassing progressive, moderate, and conservative [17]. As such, Naver.com’s comprehensive domestic usage and broad political spectrum collectively contribute to support its data resource’s validity and reliability to generalize the study results. The search terms were ‘Jungshinbunyeolbyung’ for mind split disorder and ‘Johyeonbyung’ for attunement disorder, and were automatically collected using Python. The dataset was divided into news articles dealing with ‘Jungshinbunyeolbyung’ as mind split disorder before and after the disease renaming (Jan 1, 2005 ~ Dec 31, 2010 and Jan 1, 2012 ~ Dec 31, 2018) and news articles dealing with ‘Johyeonbyung’ as attunement disorder after the disease renaming (Jan 1, 2012 ~ Dec 31, 2018) to investigate differences in the social perception between the disease names before and after the revision. Of the articles collected, overlapping articles between the different media reports were excluded from the analysis. As results, the total numbers of articles used in the analysis were 2,743 (mind split disorder before the renaming), 3,114 (mind split disorder after the renaming), and 3,068 (attunement disorder).

#### Number of patients who have admitted to psychiatric hospitals with a diagnosis code of schizophrenia

Data for each month from January 2010 to July 2018 on the number of people who have admitted to psychiatric wards with schizophrenia diagnosis (ICD-10 code F20) was collected from Korea’s healthcare big data system (http://opendata.hira.or.kr).

#### Media coverage of general crimes committed by schizophrenia patients

For media coverage, we only considered the three terrestrial TV networks in Korea (i.e., KBS, MBC, and SBS) mainly because those networks exert more significant influences on audiences than other types of media as suggested by prior studies. The number of news articles published by each TV network about general crimes committed by patients with schizophrenia each month between January 2010 and July 2018 was counted using the most popular news aggregator in Korea, which is Naver.com.

### Analysis

#### Latent dirichlet allocation topic modeling

We analyzed the social perception of the mind split disorder and attunement disorder before and after the schizophrenia renaming, by collecting online news articles related to schizophrenia and performing various text mining techniques. At First, the overall characteristics of online news articles were examined using Latent Dirichlet Allocation (LDA) topic modeling for a macroscopic language analysis. LDA is the most widely used topic modeling technique that calculates the probability distribution of infeasible terms at each of topic groups that are extracted from the article collections [18–20]. In this study, LDA topic modeling was performed to investigate the difference in the media topics related to the disease names before and after the renaming.

LDA topic modeling was performed on a dataset divided into news articles by period (before/after the revision of disease name) and by disease names (‘Jungshinbunyeolbyung’ for mind split disorder and ‘Johyeonbyung’ for attunement disorder). We tried to utilize the perflexity values to determine the analyzable number of topics, but the values decreased monotonically in all sections. Thus, in this study, we determined the analyzable number of topics as 30, which were found appropriate to interpret due to high similarity between major keywords in the same topic and low similarity between topics, after performing topic modeling by setting the number of topics to 10, 20, 30, and 40. The authors extracted 20 keywords per topic, and annotated each of the topics based on the association between keywords within the topics. To evaluate the classifications, two independent psychologists were invited to engage, and they qualitatively confirmed the reliability of the suggested terms representing the different media frames [21]. The inter-investigator agreement scores in both loose and strict matches were calculated by dividing the number of consistently agreed topics by all the topics. To further validate the inter-investigator agreement scores, we additionally adopted Krippendorff’s alpha [22] to correct any potential biases arising from the redundancy of media frames and participating number of investigators. For analysis the Gensim function [23] in Python modules was used, and the LDA topic modeling results were visualized with pyLDAvis.

#### Term frequency-inverse document frequency weight analysis

For a microscopic language analysis, the relationship and contextual features between articles were examined using Term Frequency-Inverse Document Frequency (TF-IDF) weight model [24]. The TF-IDF is a linguistic analysis approach to evaluate how important a word inside an article is for text mining. The larger the TF-IDF value, the more likely the word is to determine the topic of the article to which it belongs, thereby suggesting a measurement to extract key keywords [25]. In the TF-IDF analysis process, TF-IDF values of the top five words per article were calculated for each of the dataset. Then, the words of varying significances were arranged with descending order based on TF-IDF values, for which the top 20 words and the bottom 3 words were compared.

#### Quantitative epidemiologic analysis

We investigated the effects of the media coverage for crimes committed by schizophrenia patients on the nationwide medical use patterns of patients with the disease, by analyzing epidemiological data under a linear regression model. In order to see the relationship between the number of news articles and the changes in the number of patients admitting to psychiatric wards, we used the following regression model:

$$Daily\_patients\_change\_rate_t=\beta_0+\beta_1\,\#news\_articles_t+\beta_2\,\#news\_articles_{t-1}+\mu\,month\_effects+\eta\,year\_effects+e_{t},$$

where.

*Daily_patients_change_rate*_*t*_ reflects the change rate of the average number of people who have admitted to psychiatric wards with a diagnosis of schizophrenia per day in month *t* over that in the preceding month. For this, the number of average daily patients was first calculated by dividing the number of monthly patients with the number of days in the month. Then, the *Daily_patients_change_rate*_*t*_ was calculated as follows:

$$\left((\#\,of\,daily\_patientst-\#\,of\,daily\_patients_{t-1}\right)/\#\,of\,daily\_patients_{t-1}\ast100$$

*#news_articles*_*t*_ refers to the number of news articles published by the three TV networks about general crimes committed by schizophrenia patients in month *t*. We also included a variable representing such media coverage in the preceding month (i.e., in month *t-1*) due to the possible lagging effects of the media coverage. Regarding *month_effects*, since it is likely that the number of people admitting to psychiatric wards varies by month, the month effects were controlled for by incorporating month dummy variables into the regression model. Regarding *year_effects*, since it is possible that the value of the dependent variable increased over time, the year effects were controlled for by including year-related dummy variables. To further convince the significance of the findings from the regression model, we simultaneously controlled the month and year effects using the both dummy variables.

## Results

### LDA topic modeling

**Fig. 1:**
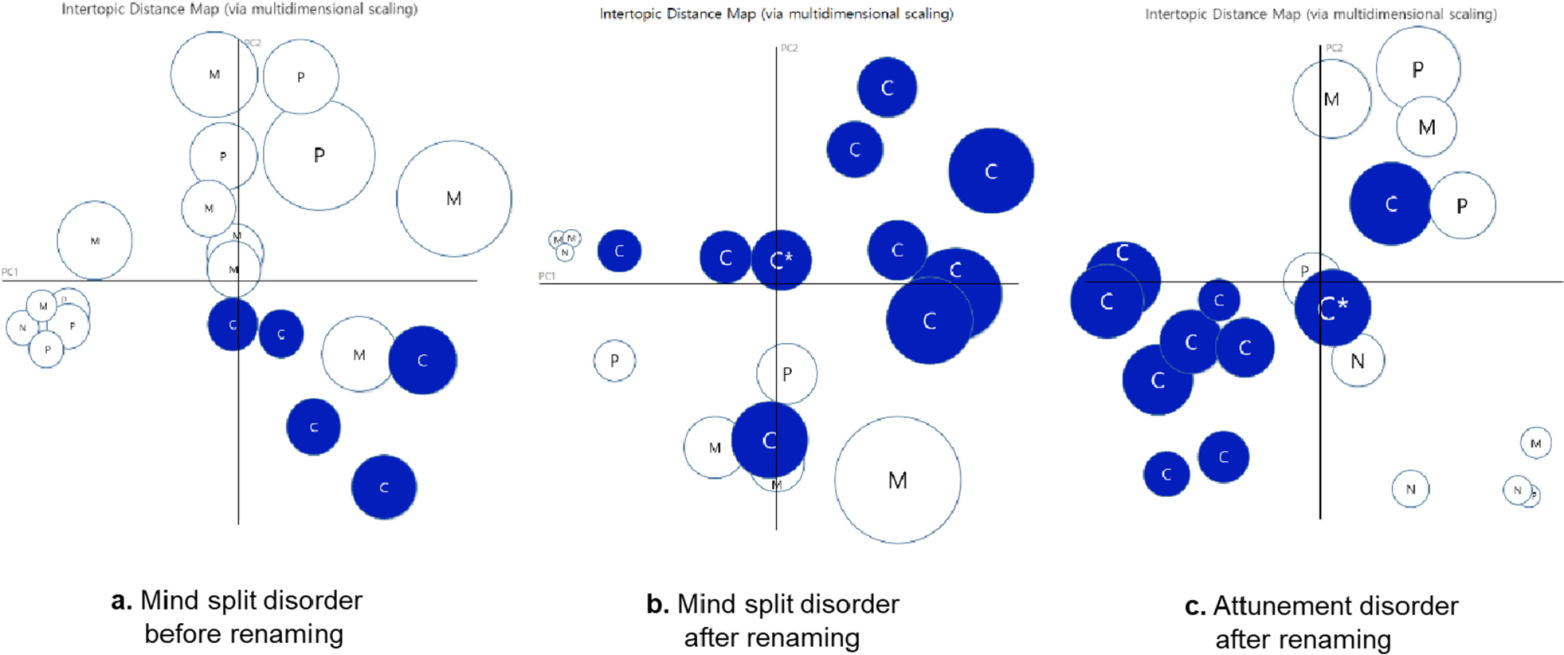
LDA topic modeling for schizophrenia before and after renaming. Principle component analysis with 2-dimension was adopted to visualize the LDA topic modeling for the disease names before and after the revision. Number (Density) of circles indicate the degree of a significance of a specific media frame within a topic. Circles with a specific media frame can be redundantly and adjacently shown in the principle component dimensions, with a trend to aggregate with each other owing to similar news keywords or contexts. **a** LDA topic modeling for ‘Jungshinbunyeolbyung’ as mind split disorder before the revision of the schizophrenia name. **b** LDA topic modeling for ‘Jungshinbunyeolbyung’ as mind split disorder after the revision. **c** LDA topic modeling for ‘Johyeonbyung’ as attunement disorder after the revision. *Abbreviations, C, Conflict frames; P, Policy frames; M, Medical frames; and N, neutral frames.* For better visualization of the conflict frames (C), blue-colored close circles were used. The conflict frame with asterisk (*) in the Fig. 1c indicates the Gangnam homicide case committed by a schizophrenia patient in 2016

The outcome of the LDA topic modeling shows the significant increase in the media topics of the conflict frames implicating schizophrenia patients in crimes regardless of the schizophrenia names (Fig. 1). The annotated topics were further estimated for their individual significance in the dataset from the perspective of media frames, by calculating the proportion of each of the topics within the dataset (Table 1). The weight of the media frame was calculated by totaling the values of topics belonging to the frame, where a value of topic was calculated by dividing the total sum of ratio scores (“theta”) [26] reflecting the significance of topic within an article by the total number of articles [27].

**Table 1:** Proportion of topics for schizophrenia by media frames

Schizophrenia name	Media frame					
	Conflict	Medical	Policy	Neutral	Miscellaneous (Politics)	Total
Mind split disorder before renaming	315 (11.5%)	1709 (62.3%)	529 (19.3%)	189 (6.9%)	-	2743 (100%)
Mind split disorder after renaming	1859 (59.7%)	786 (25.25%)	221 (7.1%)	104 (3.35%)	143 (4.6%)	3114 (100%)
Attunement disorder	1611 (52.5%)	693 (22.6%)	313 (10.2%)	252 (8.2%)	199 (6.5%)	3068 (100%)

Topics were classified into four different media frames as follows: medical frames (symptoms, research, causes, and treatments), conflict frames (crime), policy frames (policy and welfare), and neutral frames (anecdotes and art). The scores were remarkably high, regardless of the metrics (strict match = 0.9, loose match = 0.99). The Krippendorff’s alpha scores were 0.88, and thus considered reliable [22]. In particular, the loose match scores were close to 1, which shows that disagreement for the classification of media frames rarely occurred between the investigators. To note, the ‘politics’ topic of dataset exploited for Fig. 1b,c was not considered in the analysis because articles irrelevant to schizophrenia such as ‘today’s popular news’ were collected at the side of the body of the articles.

Table 1 shows that medical frames were the most common in news articles of schizophrenia (i.e. ‘Jungshinbunyeolbyung’ as mind split disorder) before the revision of the disease name, followed by policy, conflict, and neutral frames. On the other hand, after the revision of the disease name, the conflict frames were the most frequently reported in the articles of schizophrenia (i.e. ‘Jungshinbunyeolbyung’ as mind split disorder), followed by medical, policy, and neutral frames. Lastly, in the articles of the renamed schizophrenia (i.e. ‘Johyeonbyung’ as attunement disorder), the conflict frames were also overwhelmingly reported, then followed by medical, policy, and neutral frames. These results indicate that there was a significant increase in the conflict frames in the articles of schizophrenia after the revision of the disease name compared to those before the revision. Crime articles of the conflict frames, which accounted for the smallest proportion of all articles before the revision of the schizophrenia name, increased by about five times after the revision of the disease name. This seems to be due to a series of media reports of violent crimes of schizophrenic patients shortly after the revision of the disease name. For instance, a distinct topic frame, which was marked with an asterisk in the Fig. 1b,c, was associated with the Gangnam station homicide case committed by a schizophrenia patient in 2016. On the other hand, the proportion of medical frames decreased by more than a half after the revision of the disease name. This suggests that medical knowledge and information such as symptoms, research, and treatment of schizophrenia were no longer regarded as a crucial social issue after the revision of the disease name, nonetheless those used to be a major topic before the revision of the disease name.

### TF-IDF weight analysis

The larger the TF-IDF weight value of a word, the more it means that it contains a key message within the article containing the word. Table 2 shows the words with the top 20 weight values of TF-IDF for each dataset. The media frames were further analyzed for the context of contents in the articles containing the top 20 words (Table 3).

**Table 2:** Top 20 words with TF-IDF weight values for schizophrenia

TF-IDF weight (order)	Mind split disorder before renaming		Mind split disorder after renaming		Attunement disorder after renaming	
1	Medical science	0.65	Symptoms	0.8	**Police**	0.65*
2	Negotiations	0.57	Hospitalization	0.59	Counseling	0.6
3	Research	0.57	**Police**	0.58*	**Police station**	0.51*
4	Stress	0.53	**Crime**	0.56*	Psychology	0.5
5	Medication dose	0.52	Mentality	0.53	Research	0.49
6	Mentality	0.51	Woman	0.48	Schizophrenia	0.49
7	Military service	0.5	Investment	0.46	**Sentence of imprisonment**	0.47*
8	Psychology	0.48	**Trial**	0.44*	Parent	0.47
9	Suicide	0.43	Parent	0.43	Mentality	0.46
10	Psychiatry	0.43	Heredity	0.41	**Crime**	0.46*
11	Counseling	0.42	Psychology	0.41	Drug	0.45
12	Child	0.38	**Psychopath**	0.4*	Mother	0.42
13	Company	0.34	**Police station**	0.39*	Woman	0.4
14	Clinic	0.34	**Criminal**	0.35*	Psychosis	0.39
15	Mind	0.29	**Allegations**	0.32*	**Trial**	0.37*
16	Delusion	0.28	Life	0.32	Abandonment	0.37
17	**Electronic anklet**	0.27*	Dementia	0.28	Homelessness	0.32
18	Improvement	0.27	Descendant	0.2	**Murder**	0.31*
19	People	0.25	Legal invalidity*	0.14	**Allegations**	0.28*
20	Culture	0.24	Designation	0.14	Daegu	0.19

Asterisk (*) indicates the word belonging to the conflict frames (crimes). Bold indicates the conflict frames

**Table 3:** Media frames including the top 20 words with TF-IDF weight values for schizophrenia

Schizophrenia name	Media frame				
	Conflict	Medical	Policy	Neutral	Sum
Mind split disorder before renaming	1	11	3	5	20
Mind split disorder after renaming	7	5	2	6	20
Attunement disorder	7	5	0	8	20

In the analysis of articles on schizophrenia (mind split disorder) before the revision of the disease name, words of medical frames such as “Medical science”, “Research”, “Medication dose”, “Psychology”, and “Psychiatry” were found to present high TF-IDF values among the top 20 words. There was only a single word with a high TF-IDF value related to conflict frames, which is “Electronic anklet”, a forceful legal procedure for sex offenders in Korea. This shows that the public was interested in medical aspects of schizophrenia rather than negative stereotype of the disease before the revision of the disease name. However, in the analysis of articles on schizophrenia (mind split disorder) after the revision of the disease name, the words with high TF-IDF values of medical frames decreased by more than a half compared to those before the revision. In contrast, words with high TF-IDF values of conflict frames accounted for 7 of the top 20 (35%) after the revision, compared to the 1 of the 20 (5%) before the revision. Similarly, in the analysis of words on schizophrenia (attunement disorder) after the revision of the disease name, there were words of conflict frames such as “Police”, “Police station”, “Murder”, and “Crime” that occupied a considerable portion of the top 20 words with high TF-IDF values (7 of the 20, 35%). In contrast, there was a decrease in the number of words related to medical frames after the renaming (5 of the top 20 words, 25%), compared to before the renaming (11 of the top 20 words, 55%). Overall TF-IDF analyses were also presented in supplementary Tables 1, 2 and 3. These findings suggest that the public interest on schizophrenia has shifted from the medical aspects to the conflict frames, regardless of the disease renaming.

### Quantitative epidemiologic analysis

The estimation results of regression model were presented in Table 4. The results in Table 4 show that when there was more news coverage of crimes committed by an individual with schizophrenia, the number of schizophrenia patients who admitted to psychiatric hospitals increased compared to that in the preceding month (*B* = 0.32, *p* = 0.035). Not much difference between male (*B* = 0.31, *p* = 0.043) and female patients (*B* = 0.34, *p* = 0.041) was found, albeit there was a slightly larger increase among female patients. Hospitalization statistics by year of schizophrenia patients were provided in Supplementary Table 4. We performed the same analysis for the other frames such as medical, political, and cultural themes, none of which was associated with the hospitalization frequencies,

**Table 4:** Estimation results of regression model

Variables	Total change		Male change		Female change	
	*B*	*SE*	*B*	*SE*	*B*	*SE*
constant	-0.18	0.99	-0.05	0.98	-0.29	1.02
# *homicide_articles_t*	0.32*	0.17	0.31*	0.16	0.34*	0.17
# *homicide_articles_t-1*	-0.15	0.17	-0.15	0.16	-0.15	0.17
*R* ^2^	0.78		0.78		0.77	
* F* (21, 85)	14.05**		14.64**		13.25**	

*Note*: *N* = 107, * *p* < 0.05, ** *p* < 0.01. The results of the dummy variables regarding month and year effects were omitted due to space constraints

## Discussions

This study exploited the comprehensive approach of linguistic and epidemiological analyses that consistently show there was a dramatic increase in media frames of negative stereotypes of schizophrenia patients after the revision of the disease name compared to before the revision. LDA topic modeling results indicate that conflict frame (crime)-related topics accounted for the majority of the media coverages for both mind split disorder and attunement disorder after the revision of the schizophrenia name, which was in contrast to the medical frame (medicine)-related topics accounting for the majority of the media coverages for mind split disorder before the revision. TF-IDF weight analysis also shows that keywords with a high significance were mostly associated with crimes in news articles of schizophrenia for both mind split disorder and attunement disorder after the revision of the name, whereas news keywords were commonly associated with medical aspects of schizophrenia for mind split disorder before the revision. In corroboration, epidemiological analyses also show there was a significant association between the number of media coverages of negative stereotypes of schizophrenia patients and the frequency of hospital admissions of schizophrenia patients. These comprehensive findings corroborate each other to show that media frames continue to focus on negative stereotypes of schizophrenia patients and scarcely alleviated by the elaboration and efforts to revise the schizophrenia name from mind split disorder to attunement disorder in purpose of mitigating the stigmatization of the disease in Korea.

This study specifically addressed the following four conclusions for the research questions: <A > The social perception of schizophrenia patients has turned to the adverse stance keeping negative stereotypes of those patients even after the disease renaming event in 2011. <B > The social perception of keeping negative stereotypes of schizophrenia patients was similarly reported between mind split disorder as ‘Jungshinbunyeolbyung’ (before the renaming of schizophrenia) and attunement disorder as ‘Johyeonbyung’ (after the renaming of schizophrenia). <C > There was a significant association between the nationwide hospital admission patterns of schizophrenia patients and the frequencies of media coverages negatively deciphering schizophrenia patients. For the changes of admission frequencies, the reasons possibly encompass the number of psychiatric beds available, and amendments in health insurance policies and mental health regulations while we were not able to find the relevant social evidence.

From the overall results, we found that the revision of the schizophrenia name from mind split disorder to attunement disorder hardly improved social perception. This is because the medical frames conveying accurate information of schizophrenia and contributing to resolving prejudice in media reports were identified to have decreased, and instead the conflict frames hampering social fear and prejudice of the disease have increased. Although the disease name was revised in purpose to eliminate the stigmatization, the social perception of schizophrenia patients has rather deteriorated by a series of unrefined reports for violent crimes. However, it needs a caution to conclude the negative perceptions have increased simply because of media reports. In fact, prejudice and discrimination against mentally ill people have long existed in both East and West [2, 28–32] because individual perceptions of mental illness are influenced by various factors, including demographic characteristics. Also, the media has been used since the 1990s to resolve prejudice against schizophrenia in the West, and there are several cases that have proven effective, indicating that the impact is not small. Thus, this study focused on the social impact per se of media frames, which can be either good or bad in building an awareness of schizophrenia.

### Limitations

The limitation of this study is that some medical jargon or neologisms may have been omitted from the analysis results because the morpheme analyzer does not reflect both proper and synthetic nouns, and the subjectivity of the researcher was inevitably reflected when labeling the topic when performing the LDA topic modeling. Future studies are required to more efficiently extract nouns using a linguistic analysis technique such as named entity recognition. Nonetheless, this study comprehensively analyzed the contents of long-term schizophrenic articles including both terrestrial news sources using text mining techniques in Korea. In addition, this study combined various text mining techniques with predefined news frame types to statistically and visually analyze large amounts of article data according to specific criteria and compensate for the limitations of objectivity that have been raised in traditional qualitative content analysis by further performing the epidemiologic analyses. On the one hand, there were various factors including psychiatric beds available and possible changes in health insurance policies and mental health regulations that could have influenced changes in the number of hospitalizations for psychiatric patients, but such data were not sufficiently reflected.

## Conclusions

This study shows the consistent findings that social perception of schizophrenia has maintained the negative stereotypes of those patients who are described as being implicated in violent crimes. This negative media frame on schizophrenia patients associated with the adverse impact on the general medical use patterns of nationwide schizophrenia patients. Of note, the effort of the revision of the schizophrenia name from mind split disorder to attunement disorder was found diluted by those negative media frames keeping the stereotypes of schizophrenia patients. Thus, the study findings support that the collaborative approaches of establishing the unbiased media frames and taking a strategy to the disease renaming are both required to improve the social perception of schizophrenia while minimizing the stigmatization.

### Supplementary Information

**Additional file 1: Supplementary Table 1.** TF-IDF analysis for mind split disorder before the revision of the schizophrenia name

**Additional file 2: Supplementary Table 2.** TF-IDF analysis for mind split disorder after the revision of the schizophrenia name

**Additional file 3: Supplementary Table 3.** TF-IDF analysis for attunement disorder after the revision of the schizophrenia name

**Additional file 4: Supplementary table 4.** Hospitalization statistics of schizophrenia patients in South Korea

## Data Availability

Data used for this study are available to any researchers upon a reasonable request with an approval from Yu Sang Lee, the corresponding author.
